# Sperm Recovery and IVF after Testicular Sperm Extraction (TESE): Effect of Male Diagnosis and Use of Off-Site Surgical Centers on Sperm Recovery and IVF

**DOI:** 10.1371/journal.pone.0069838

**Published:** 2013-07-29

**Authors:** Kenan Omurtag, Amber Cooper, Arnold Bullock, Cathy Naughton, Valerie Ratts, Randall Odem, Susan E. Lanzendorf

**Affiliations:** 1 Division of Reproductive Endocrinology and Infertility, Department of Obstetrics and Gynecology, Washington University, St Louis, Missouri, United States of America; 2 Department of Urology, Washington University, St Louis, Missouri, United States of America; 3 Metropolitan Urological Specialists, St Louis, Missouri, United States of America; Clermont-Ferrand Univ., France

## Abstract

**Objective:**

Determine whether testicular sperm extractions and pregnancy outcomes are influenced by male and female infertility diagnoses, location of surgical center and time to cryopreservation.

**Patients:**

One hundred and thirty men undergoing testicular sperm extraction and 76 couples undergoing 123 in vitro fertilization cycles with testicular sperm.

**Outcome Measures:**

Successful sperm recovery defined as 1–2 sperm/0.5 mL by diagnosis including obstructive azoospermia (n = 60), non-obstructive azoospermia (n = 39), cancer (n = 14), paralysis (n = 7) and other (n = 10). Obstructive azoospermia was analyzed as congenital absence of the vas deferens (n = 22), vasectomy or failed vasectomy reversal (n = 37) and “other”(n = 1). Sperm recovery was also evaluated by surgical site including infertility clinic (n = 54), hospital operating room (n = 67) and physician’s office (n = 11). Treatment cycles were evaluated for number of oocytes, fertilization, embryo quality, implantation rate and clinical/ongoing pregnancies as related to male diagnosis, female diagnosis, and use of fresh or cryopreserved testicular sperm.

**Results:**

Testicular sperm recovery from azoospermic males with all diagnoses was high (70 to 100%) except non-obstructive azoospermia (31%) and was not influenced by distance from surgical center to laboratory. Following in vitro fertilization, rate of fertilization was significantly lower with non-obstructive azoospermia (43%, p = <0.0001) compared to other male diagnoses (66%, p = <0.0001, 59% p = 0.015). No differences were noted in clinical pregnancy rate by male diagnosis; however, the delivery rate per cycle was significantly higher with obstructive azoospermia (38% p = 0.0371) compared to diagnoses of cancer, paralysis or other (16.7%). Women diagnosed with diminished ovarian reserve had a reduced clinical pregnancy rate (7.4% p = 0.007) compared to those with other diagnoses (44%).

**Conclusion:**

Testicular sperm extraction is a safe and effective option regardless of the etiology of the azoospermia. The type of surgical center and/or its distance from the laboratory was not related to success. Men with non-obstructive azoospermia have a lower chance of successful sperm retrieval and fertilization.

## Introduction

Since its introduction in 1992, in vitro fertilization (IVF) with intracytoplasmic sperm injection (ICSI) has proved to be the only viable option for patients with azoospermia desiring their own genetic offspring. [1], [2] Sperm can be obtained directly via the epididymis, either percutaneously (PESA) or microsurgically (MESA), or by direct sampling of the testis either via needle aspiration (TESA) or direct biopsy (TESE). When extracted from the testes, sperm have not undergone full maturation compared to epididymal or vasal sperm. Ultimately, the location and method of sperm extraction does not affect pregnancy rate. [3]–[6].

Obstructive azoospermia accounts for 40% of men who present to infertility practices with azoospermia and is often successfully treated with ICSI using surgically collected sperm. [7] Men with non-obstructive azoospermia (NOA) may have severely deficient spermatogenesis and inadequate sperm production. [8] Non-obstructive azoospermia is most frequently characterized by an elevated FSH and small volume testes, and treatment historically involved donor inseminations or adoption. Sperm production is not uniform throughout the testes in men with NOA which leads to difficulty in surgically isolating sperm. However, if sperm are recovered, subsequent IVF-ICSI treatment can produce viable offspring for these patients. [8].

Testicular sperm extraction may also be indicated in patients recently diagnosed with cancer due to deficient sperm in the ejaculate or an inability to collect. Alkylating agents are the most toxic to Leydig and Sertoli cells leading to decreased testicular volume and increased FSH. [9] In addition, accidents and critical illnesses, along with requests for posthumous reproduction are increasing the need for TESE procedures. [10], [11]The ability to cryopreserve sperm after testicular extraction allows better coordination with oocyte retrieval.While cryopreservation of testicular sperm is equal to fresh, even among some cohorts of men with NOA [12]–[16], concern regarding limited sperm viability during the freeze-thaw in men with NOA [8] remain prompting some to perform TESE on the day of egg retrieval to avoid sperm loss from the freeze-thaw [17].

While the diagnosis itself has remained stable, male factor infertility remains the most frequent diagnosis among IVF cycles performed by Society of Assisted Reproductive Technologies (SART) Member Clinics and overall IVF utilization continues to rise. [18] In an effort to optimize patient scheduling, it is not uncommon for surgeons to perform TESE procedures outside the vicinity of the IVF laboratory, thus requiring transportation of the fresh specimen to the laboratory for ICSI or cryopreservation. Over a 15 year period, our TESE’s were performed at three different locations at varying distances from the IVF laboratory. Additionally, we have had two urologists performing the procedures over that time period.

We present our institutional experience over the last fifteen years as it relates to IVF-ICSI using TESE sperm. We examined our sperm recovery rate based on male factor infertility diagnosis and IVF-ICSI outcomes comparing fresh and frozen TESE sperm. Cycle outcomes (i.e pregnancy rate and live birth rate) were correlated with male factor diagnosis, as well as co-existing female factor infertility. In addition, we present data on outcomes as they relate to the location and surgical provider.

## Materials and Methods

### Patient Selection

This study was approved by the Human Studies Committee of Washington University School of Medicine and included all TESE procedures and subsequent IVF-ICSI cycles performed in our Division from January 1995 through December 2009 (Table 1). The Human Studies Committee agreed that it was a record review and did not pose a risk to the participants since all data was de-identified.

**Table 1:** Diagnosis, age and cryopreservation results for patients undergoing testicular sperm extraction.

Diagnosis	n	Mean age ±SD	No. with samples cryopreserved (%)
**Obstructive Azoospermia**	**60**	**39.8**±**8.2**	**60 (100)**
Congenital Absence of Vas Deferens	22	33.4±7.8^a^	22 (100)
Vasectomy/Failed Vasectomy Repair	37	43.4±6.0^a^	37 (100)
Other	1	46	1 (100)
**Non-Obstructive Azoospermia**	**39**	**36.0**±**6.8**	**12 (31)**
High FSH	4	34.5±8.3	2 (50)
Sertoli Cell Only	6	35.0±3.2	2 (33)
Maturation Arrest	8	35.6±7.0	2 (25)
Spermatogenic hypoplasia	1	31.0	0
Unknown	20	36.9±7.8	6 (30)
**Cancer**	**14**	**36.6**±**11.5**	**10 (71)**
Testicular	6	30.0+3.6^b^	3 (50)
Prostate	2	53.5+3.5^b^	2 (100)
ALL	1	31	1(100)
Bladder	1	63	1 (100)
Hodgkin’s	1	35	1 (100)
Type not known	3	31.7+2.5^b^	2 (67)
**Paralysis**	**7**	**35.0+7.0**	**7 (100)**
**Other**	**10**	**34.4+3.7**	**10 (100)**
Kidney transplant	1	27.0	1 (100)
Autoimmune disease	1	38.0	1 (100)
Stroke	1	39	1 (100)
Testicular torsion	2	32.5+2.1	2 (100)
Varicocoele/varicocoele repair	3	34.0+1.0	3 (100)
Anejaculation	1	34	1 (100)
Electrocution	1	39	1 (100)

a p = 0.0001;

b = 0.0001.

TESE surgical procedures were performed by two urologists at three different locations: 1) operating room (OR) adjacent to laboratory, 2) OR in another building approximately 0.74 miles from laboratory, and 3) Ambulatory surgical center (ASC) approximately 15 miles from the laboratory. At the second site, an embryologist was present during the surgery to make an initial examination of the tissue for the presence of spermatozoa and transport the specimen to the laboratory for final processing. There was no embryologist at the third location, and patients were responsible for prompt transportation of the specimen, either with a family member or a courier, to the laboratory where the embryologist would review the sample. Couriers were instructed to keep the specimen at body temperature during transport. The time to transport from the third site was less than 30 minutes.

### TESE Surgery

Prior to presenting for a TESE, the men were required to have a negative screen for HIV, hepatitis B and C and syphilis (RPR). Following IV sedation, a small area of the scrotum is shaved, prepped and outlined in a sterile field. A testicle was held by the surgical assistant under the median raphe, and 1% lidocaine was injected as a local anesthetic. A small (5 mm) incision was made longitudinally, and the incision carried down through the fascia with a bovie cautery. The tunical vaginalis was exposed and opened, and an ophthalmic clamp placed as a wound retractor. With the testicle exposed, a 6.0 prolene suture was placed through the tunica albuginea at the upper pole, anterior aspect of the testicle as a stay suture. Microscissors or a #15 blade scalpel was used to make a 2 mm incision and testicular tissue was expressed by manipulating (squeezing) the gland. A small amount of tissue was excised. This tissue was immediately placed in culture medium, minced with sterile scissors, dispersed between two glass slides, and studied under the microscope by an embryologist. While the sample from the first site was being studied, one to two other sites would be similarly biopsied. When a biopsy site yielding spermatozoa was identified, additional tissue (10–15 mg) was harvested. The biopsy sites were closed with 6.0 prolene and the wound irrigated with saline prior to approximation of the fascia with 3.0 chromic. The skin was closed with 4.0 chromic using a simple, running stitch. A gauze dressing was applied and the patient awakened.

### Preparation and Cryopreservation of TESE Samples

Upon arrival to the laboratory, tissue was cut into 1 mm^2^ blocks in HEPES-HTF (Irvine Scientific, Santa Ana, CA)+5% Human Serum Albumin Solution (HSA; Irvine Scientific) followed by mincing with scissors or the bottom of a sterile 5 ml tube (Falcon 2058, Beckton Dickenson, Franklin Lakes, NJ). Periodically, a small portion of the minced fluid was examined under the inverted microscope (Olympus Inc., Center Valley, PA 18034) for the presence of spermatozoa. Approximately 0.5 ml aliquots of fluid and minced tissue found to contain spermatozoa (motile and/or non-motile) were transferred to cryovials (Nalge Nunc Intl., Rochester, NY) for subsequent cryostorage. All processing was performed in a sterile hood at room temperature and processing results were reported as the number of motile and/or nonmotile spermatozoa seen per 20X microscopic field. Processed samples containing no spermatozoa were not cryopreserved. If the sample or a portion of the sample was to be used fresh for ICSI, it was processed as described below and stored at 37°C, 5% CO_2_ until the time of the procedure.

Cryopreservation of TESE samples was performed by adding an equal volume of Test yolk buffer (TYB) with 12% glycerol (Irvine Scientific) drop wise at a rate of 1/4th the original sample volume every 5 minutes. After insuring sufficient mixing of TYB and sample, cryovials were incubated at 2.0°C for 60 minutes, followed by slow cooling in liquid nitrogen vapors using a Taylor-Warton Freezing rack (Praxair Dist. Inc., St Louis, MO). Following freezing, cryovials were stored in liquid nitrogen until time of thaw.

### Ovarian Stimulation and Oocyte Retrieval Procedures

Ovarian stimulation was performed using gonadotropins in combination with a GnRH antagonist or GnRH agonist following established protocols. Serum estradiol was measured at baseline and serially after gonadotropin administration,depending on the stimulation protocol and then as needed until retrieval. Vaginal ultrasounds were performed concomitantly with estradiol levels. Human chorionic gonadotropin (hCG)was administered when two or more follicles were at least 17–18 mm in largest mean diameter. Transvaginal follicular aspiration was performed approximately 36 hours later (day 0).

### Preparation of TESE Samples for Intracytoplasmic Sperm Injection (ICSI)

Preparation of cryopreserved TESE samples for ICSI was performed the day of the oocyte retrieval. One or more cryovials were removed from liquid nitrogen and thawed in a 31°C water bath. The sample was then washed twice by centrifugation in approximately 3 to 5 ml of IVC-TWO (In Vitro Care Inc., Frederick, MD) supplemented with 5% HSA. The final pellet was resuspended in 0.1 to 0.2 ml of IVC-TWO+5% HSA and incubated under oil at 37°C, 5% CO_2_. If at the time of ICSI, no or few motile sperm were present in the sample, an equal amount of 2 mM pentoxifylline (Sigma Chemical Co., St. Louis, MO) in HEPES-HTF supplemented with 10% Synthetic Serum Substitute (SSS, Irvine Scientific) was added to promote motility. Fresh TESE samples were washed as described above and were sometimes collected the day prior to oocyte retrieval and incubated overnight for increased sperm motility. When necessary, pentoxifylline was also used with the fresh TESE samples if no or poor motility was found at the time of ICSI.

### Intracytoplasmic Sperm Injection (ICSI) and Embryo Culture

Oocyte identification and oocyte and embryo culture were performed using established protocols for in vitro fertilization and intracytoplasmic sperm injection. Single oocytes, cultured in 0.1 mL droplets of IVC-TWO+5% SSS were injected with a single sperm. Whenever possible, motile or twitching sperm with a normal morphology were utilized. Injected oocytes were evaluated approximately 18 hours later for pronuclear formation, and the resulting embryos placed in 0.05- mL droplets of IVC-ONE growth medium (In Vitro Care Inc.) supplemented with 10% SSS. Embryo cleavage and morphology was evaluated again on the morning of day 3. Embryos remaining in culture for a day 5 transfer or for possible cryopreservation at the blastocyst stage were transferred to 0.05–mL droplets of IVC-THREE growth medium (In Vitro Care Inc.) supplemented with 10% SSS.

### Embryo Transfer

Embryo transfers were performed on the morning of day 3 or day 5 depending on the number of good quality embryos. When multiple embryos were available, the Veeck criteria were used to select the highest grade embryos for transfer. [19] All efforts were made to follow American Society of Reproductive Medicine (ASRM) embryo transfer guidelines. [20].

All transfers, regardless of day, were performed using a Wallace catheter (Irvine Scientific) with the patient in the dorso-lithotomy position with a full bladder. Patients were instructed to remain resting on their backs for at least 30 minutes following the embryo transfer. Progesterone in oil was administered on the day of retrieval and then daily for 8 weeks unless a negative beta-hCG was obtained.

All patients underwent an initial serum test for beta-hCG 12 to 14 days after embryo transfer. A clinical pregnancy was defined as the presence of a gestational sac on ultrasound.

### Statistical Analysis

Cycle demographics and treatment cycle variables were compared using either a Kruskal-Wallis test or a one-way analysis of variance. Proportions were analyzed with a chi-squared or Fisher’s exact test. *P-values* <0.05 were considered statistically significant (GraphPad InStat version 5.1, GraphPad Software, San Diego CA).

## Results

One-hundred and thirty testicular sperm extraction (TESE) procedures were performed at our center from January 1995 through December 2009. The most common diagnosis for TESE among men at our center was obstructive azoospermia (46%). Non-obstructive azoospermia (24%) and cancer (11%) followed in frequency.

The average age of these men was thirty-six. Statistically significant differences in age, however, were reached between men with congenital bilateral absence of the vas deferens (CBAVD) and men with a history of vasectomy (p = 0.0001). Additionally, men diagnosed with testicular cancer were younger than men with prostate cancer (p = 0.0001).

Recovery of sperm via TESE was 70% in cancer patients and 100% in men diagnosed with obstructive azoospermia, paralysis and other. Thirty-one percent of men diagnosed with NOA had sperm available after TESE. (Table 1).

In evaluating the effect of surgeon and surgery site on successful recovery and cryopreservation of sperm, there was no statistically significant difference between the locations as far as a) patient age, b) number of patients with non-obstructive azoospermia, and c) number of samples cryopreserved. Additionally, when comparing the two different surgeons at our facility, no statistically significant differences in these three categories were found (Table 2).

**Table 2:** Effect of distance of surgical suite from laboratory and surgeon on sperm recovery and cryopreservation of TESE samples.

Site^a^/MD	n	Mean age±SD	No. of cases with NOA (%)	No. samples cryopreserved (%)
Site 1 (0 miles)	54	36.7±8.4	30 (55)	40 (74)
Site 2 (0.74 miles)	67	38.6±7.5	39 (58)	49 (73)
Site 3 (15 miles)	11	35.5±9.0	3 (27)	10 (91)
Urologist 1	86	37.9±7.9	42 (49)	64 (74)
Urologist 2	46	37.1±8.3	30 (65)	35 (76)

a (distance from surgical suite from IVF laboratory).

Note: NOA = non-obstructive azoospermia.

Of the men who had TESE performed, 58% (76/130) went on to use the sample in an in-vitro fertilization cycle. Of these 76 men, those diagnosed with obstructive azoospermia (55%) comprised the majority while 25% of those with other diagnoses (i.e cancer, paralysis, testicular torsion, varicocele, etc) and 20% of men with non-obstructive azoospermia were involved in an IVF cycle. The mean age of the female partner was thirty three. The fertilization rate was greater in the obstructive azoospermia group compared with the non-obstructive azoospermia group (66% vs 43%, p = <0.0001). Additionally, the delivery rate in men undergoing IVF with obstructive azoospermia was higher than men with non-obstructive azoospermia (62% vs. 20%; p = 0.007)(Table 3).

**Table 3:** Effect of Male Diagnosis on TESE/IVF Outcome.

	Obstructive	Non-Obstructive	Other^a^	Total
No. Retrievals	72	19	32	123
No. Patients	42	15	19	76
Mean No. 2PN	6.2±0.50^b^	4.1±0.95^b^	4.6±0.57	5.5±0.37
Fertilization Rate	439/670 (66%)c,d	77/179 (43%)c,e	142/257 (55%)d,e	658/1106 (59%)
Implantation Rate	48/167 (29%)	7/27 (26%)	13/65 (20%)	68/259 (26%)
Delivery Rate per ET (all patients)	26/68 (38%)^f^	3/14 (21%)	5/30 (16.7%)^f^	34 (30.4%)
Delivery Rate per Patient	26/42 (62%)g,h	3/15 (20%)^g^	5/19 (26.3%)^h^	34/76 (44.7%)
Mean age of Female partner (range)	33.3±0.60 (24–43)	33.8±0.93 (26–40)	31.6±0.84 (23–43)	33.1±0.44 (23–43)

a includes all male diagnoses except obstructive and non-obstructive azoospermia.

b p = 0.044;

c p<0.0001;

d p = 0.049;

e p = 0.015;

f p = 0.0371;

g p = 0.0070;

h p = 0.0134.

Men who had CBAVD had significantly younger female partners (mean age 30.5, p = 0.0001) than men who had a prior vasectomy (mean age 35.5, p = 0.0001). The associated embryo implantation rate was higher in this group of men (41%, p = 0.009) compared to that associated with men who had a prior vasectomy (21%, p = 0.009). The delivery rate per patient differed (67% vs 61%) in men with CBAVD and prior vasectomy respectively, but did not reach statistical significance (Table 4).

**Table 4:** Comparison of IVF Outcome between TESE for Congenital Absence of Vas and Vasectomized/Failed Vasectomy Reversals.

	CAV	Vasectomy
No. Retrievals	29	42
No. Patients	18	23
Mean No. Mature Oocytes	11.8±1.2^a^	8.3±0.60^a^
Mean No. 2PN	8.0+0.91^b^	5.1+0.49^b^
Fertilization Rate	230/341 (67%)	215/349 (62%)
Mean No. Embryos Transferred	2.2±0.18	2.5±0.18
No. Cycles with ET	28	40
Implantation Rate	26/64 (41%)^e^	22/103 (21%)^e^
Clinical Pregnancies per ET	14/28 (50%)	14/40 (35%)
Delivery Rate per ET	12/28 (43%)	14/40 (35%)
Delivery Rate per Patient	12/18 (67%)	14/23 (61%)
Mean age of Female partner (range)	30.5±0.77^c^ (24–38)	35.5±0.72^c^ (26–43)
Mean age of Male partner (range)	34.7±1.5^d^ (25–54)	44.9±0.85^d^ (33–55)

a p = 0.0055;

b p = 0.0047;

c,d p = 0.0001;

e p = 0.0088.

We also examined the effect of female infertility on TESE/ICSI cycles at our center. Seventy-seven women underwent 122 retrievals involving a TESE/ICSI cycle. Of these women, 65% (50/77) had no contributing female infertility factor, 19% (15/77) had diminished ovarian reserve and 17% (12) had other contributing female infertility factors (i.e endometriosis, tubal factor, PCOS, ovulatory dysfunction). The implantation rate was 33.7%, 5.3%, and 25% in the three groups, respectively. The delivery rate per patient was 54%, 6.7% and 41.7%, respectively. These differences were all statistically significant (p<0.05) (Table 5).

**Table 5:** Effect of Female Infertility on IVF/TESE Cycles.

	No Known Contributing Female Infertility	Plus Female Infertility^a^	DOR	
No. Retrievals	72	19^b^	31	
No. Patients	50	12	15	
Mean No. Mature Oocytes	11.0±0.56^c^	9.4±1.1^d^		4.8±0.61^c,^ d
Mean No. 2PN	6.8±0.50^e^	4.7±0.76^f^	2.9±0.44e,f	
Fertilization Rate	480/792 (60.6%)^g^	90/178 (50.6%)^g^	86/149 (57.7%)	
Mean No. Embryos Transferred	2.4±0.13	2.1±0.23	2.0±0.17	
No. Cycles with Embryo Transfer	68	16	27	
Implantation Rate	55/163 (33.7%)^k^	9/36 (25%)^l^	3/57 (5.3%)^ k,^ l	
Clinical Pregnancies per Embryo Transfer	33/68 (48.5%)^l^	7/16 (44%)^n^	2/27 (7.4%)l,n	
SABs per ET	6/68 (8.8%)	2/16 (12.5%)	1/27 (3.7%)	
Multiple Rate per clinical Pregnancy	19/33 (57.5%)	2/7 (28.6%)	1/2 (50%)	
Delivery Rate per ET	27/68 (39.7%)^o^	5/16 (31.3%)^p^	1/27 (3.7%)o,p	
Delivery Rate per Patient	27/50 (54%)^q^	5/12 (41.7%)	1/15 (6.7%)^q^	
Mean age of female partner (range)	31.8±0.55^h^ (23–43)	33.5±1.3 (27–43)	36.0±0.59^ h^ (29–42)	
Mean age of male partner (range)	37.1±0.85^i^ (25–63)	38.6±1.7^j^ (27–48)	45.1±1.4^ i,^ j (33–56)	

a excludes Diminished Ovarian Reserve (DOR) patients.

b female diagnoses included endometriosis (1 retrieval), tubal (7), adhesions (2), PCO (2), and ovulation disorder (7).

c,e,h,i,k,m p<0.0001;

d p<0.0003;

f p<0.0002,

e p = 0.0147;

j p = 0.0048;

l p = 0.0094;

n p = 0.0078;

o p = 0.0003;

p p = 0.0207;

q p = 0.001.

The number of in-vitro fertilization initiated retrievals that utilized either fresh or frozen TESE spermatozoa was one hundred and thirty-six. Of these retrievals, 84% (114/136) involved frozen TESE sperm and the remaining 16% used fresh sperm. A statistically significant difference in fertilization rate was noted between frozen (62%) and fresh (47%) sperm, respectively (p = 0.0003) There was no difference between the delivery rate. (Table 6).

**Table 6:** Comparison of IVF Cycles using either Frozen or Fresh TESE Samples.

	Frozen TESE Sample	Fresh TESE Sample
No. Retrievals	114	21
No. Retrievals with Obstructive Azoospermia	70 (61.4%)	2 (9.5%)
Mean No. Mature Oocytes	9.0±0.50	10.4±1.1
Mean No. 2PN	5.6±0.40	4.9±0.88
Fertilization Rate	585/945 (61.9%)^a^	78/167 (46.7%)^a^
Mean No. Embryos Transferred	2.3±0.10	2.5±0.24
No. Cycles with Embryo Transfer	98 (86%)	13 (62%)
Implantation Rate	58/225 (25.8%)	8/33 (24.2%)
Clinical Pregnancies per Embryo Transfer	38/98 (38.8%)	5/13 (38.5%)
Delivery Rate per ET	30/98 (30.6%)	4/13 (30.8%)
Mean age of Female partner	33.3±0.49	32.0±0.90
Mean age of Male partner	39.9±0.84	36.6±1.2

a p = 0.0003.

## Discussion

Previous reports on sperm recovery rates from either the epididymis or testes have ranged from 45 to 73%. [16], [21]–[27] In men with NOA, the success rates have been significantly lower, from 25–50% [21], [22], [28], but are improved when increasing the sample area [29]–[31]. Our experience using fresh and frozen TESE sperm in ICSI cycles was consistent with prior reports in regard to sperm recovery and fertilization outcomes. [12], [13], [32] Unlike previous studies that focused on fresh versus frozen TESE sperm in obstructive azoospermia, in this report we included all male factor diagnoses, including non-obstructive azoospermia.

Ambulatory surgery centers have become much more common in the United States due to advances in surgical procedures and anesthesia. [33] In 2006, there were 4,700 ASCs in the United States up from 2,462 in 1997. [34] One unique aspect of our study was the incorporation of location and surgeon information into the outcomes data as it related to cryopreservation. This has not been previously reported, and it is of interest as many IVF practitioners utilize an offsite urologist for the TESE. We did not find that the location of the TESE procedure or the urologist performing the procedure contributed to any difference in the ability to cryopreserve sperm for later coordination with oocyte retrieval. This data should provide reassurance to those practitioners and laboratory directors that may have concerns regarding the use of an offsite location for testicular extraction.

The use of spermatozoa retrieved from testes in men with azoospermia with subsequent fertilization of oocytes via ICSI was reported in 1994. [35] Rates of oocyte fertilization with TESEICSI have been reported to be between 45%–82%. [25]–[27], [35], [36] When comparing men with two common types of obstructive azoospermia (congenital bilateral absence of the vas deferens and prior vasectomy) the only difference was in their age with men with a prior vasectomy as well as their female partners being significantly older than their CBAVD counterparts. We found no difference in pregnancy rate among these two subgroups.

In men with NOA, sperm recovery and fertilization remains challenging, with lower recovery (60–70%) and fertilization rates (47–55%) compared to men with obstructive azoospermia. [8], [16], [37]–[39] In our laboratory, we documented a significantly higher rate of fertilization among men with obstructive azoospermia compared to men with NOA. Our findings are consistent with other experts who note lower sperm freeze-thaw rates with NOA than other etiologies. Ultimately, for men with NOA, the preferred method of sperm retrieval involves microdissection TESE.[22], [40]–[42]Intracytoplasmic sperm injection with sperm from TESE has radically changed the options available to these men.

Another observation worthy of notice was the effect of underlying female factor infertility on TESE/ICSI outcome particularly as it relates to pregnancy outcome. In our experience, men with azoospermia whose partners had diminished ovarian reserve had fertilization rates with TESE similar to those men whose partners had no known contributing female factor. However, implantation rate and delivery rate were significantly reduced in the group of patients that had contributing DOR (Table 5). Not surprisingly, women in the DOR group were older than those females with no known contributing female factor. These findings reinforce the challenges in achieving pregnancy in women with DOR, and although the fertilization rates may be similar, pregnancy rates remain poor with concomitant sever male factor. p.

Studies have indicated no difference in pregnancy rates in oocytes fertilized with fresh TESE sperm versus frozen-thawed TESE sperm. [13], [16], [43]–[47] Advantages of the latter method include 1) presence of reliable sperm at the time of oocyte retrieval and 2) avoiding repeat biopsy which may threaten testicular function in men with NOA. [48] Others however, suggest that the use of fresh sperm after TESE in IVF-ICSI cycles yields better fertilization and pregnancy rates. [49], [50] In males with all diagnoses frozen TESE sperm resulted in a better fertilization rate compared to fresh TESE sperm. [47] Pregnancy rates were similar between the two groups, though. In a subgroup analysis of men with non-obstructive azoospermia, there was no difference in pregnancy rate between the use of fresh and frozen-thawed TESE sperm [32].

The use of intracytoplasmic sperm injection and the evolution of testicular extraction of sperm have provided an opportunity for men to have their own biological child. Previously, these men had no options. TESE-ICSI has been a part of modern day infertility practice for 15 years and cryopreservation techniques have provided reliable coordination with oocyte retrieval. Despite the advances, azoospermia remains a challenging component of male factor infertility.
